# Automated classification of clinical trial eligibility criteria text based on ensemble learning and metric learning

**DOI:** 10.1186/s12911-021-01492-z

**Published:** 2021-07-30

**Authors:** Kun Zeng, Yibin Xu, Ge Lin, Likeng Liang, Tianyong Hao

**Affiliations:** 1 School of Data and Computer Science, Sun Yat-Sen University, Guangzhou, China; 2 National Engineering Research Center of Digital Life, Sun Yat-Sen University, Guangzhou, China; 3 School of Computer Science, South China Normal University, Guangzhou, China

**Keywords:** Eligibility criteria classification, Metric learning, Focal loss, Ensemble learning, Clinical trial

## Abstract

**Background:**

Eligibility criteria are the primary strategy for screening the target participants of a clinical trial. Automated classification of clinical trial eligibility criteria text by using machine learning methods improves recruitment efficiency to reduce the cost of clinical research. However, existing methods suffer from poor classification performance due to the complexity and imbalance of eligibility criteria text data.

**Methods:**

An ensemble learning-based model with metric learning is proposed for eligibility criteria classification. The model integrates a set of pre-trained models including Bidirectional Encoder Representations from Transformers (BERT), A Robustly Optimized BERT Pretraining Approach (RoBERTa), XLNet, Pre-training Text Encoders as Discriminators Rather Than Generators (ELECTRA), and Enhanced Representation through Knowledge Integration (ERNIE). Focal Loss is used as a loss function to address the data imbalance problem. Metric learning is employed to train the embedding of each base model for feature distinguish. Soft Voting is applied to achieve final classification of the ensemble model. The dataset is from the standard evaluation task 3 of 5th China Health Information Processing Conference containing 38,341 eligibility criteria text in 44 categories.

**Results:**

Our ensemble method had an accuracy of 0.8497, a precision of 0.8229, and a recall of 0.8216 on the dataset. The macro F1-score was 0.8169, outperforming state-of-the-art baseline methods by 0.84% improvement on average. In addition, the performance improvement had a p-value of 2.152e-07 with a standard t-test, indicating that our model achieved a significant improvement.

**Conclusions:**

A model for classifying eligibility criteria text of clinical trials based on multi-model ensemble learning and metric learning was proposed. The experiments demonstrated that the classification performance was improved by our ensemble model significantly. In addition, metric learning was able to improve word embedding representation and the focal loss reduced the impact of data imbalance to model performance.

## Background

A clinical trial is any systematic study of a test drug or treatment in humans to confirm or reveal the effects and adverse effects of the drug or treatment with the goal of determining the efficacy and safety. Eligibility criteria are established by the investigators of clinical trials and are used to identify compliance of participants with the main criteria of clinical trials [1]. Recruitment of clinical trial subjects is generally processed by manually comparing medical records with eligibility criteria [2], which is time-consuming and cost-sensitive [3]. Therefore, clinical trials commonly face difficulties during recruitment, such as participant mismatch, long recruitment cycles, and subject attrition [4]. In addition, eligibility criteria text is usually short and informally represented with a feature-sparse issue. Therefore, the construction of an automatic method using natural language processing (NLP) techniques to effectively classify clinical trial eligibility criteria text is still a challengeable research [5, 6].

Unlike other domain text, the peculiarities of medical text makes this domain text poorly classified. First, medical text has a large number of domain-specific terms. For example, the names of diseases, drugs, body parts, and other medical terminology information, so existing text segmentation methods are not applicable to such text and effective text feature extraction is difficult [7]. Secondly, medical text has a diversity of terms [8]. For example, a disease concept may have more than 10 different names in an entire dataset [8]. In addition, medical text data generally suffer from data imbalance, which makes model classification and subsequent label prediction difficult [9]. Finally, less research has been conducted on eligibility criteria, mainly involving information extraction [10–12], and less research has been conducted on classification, with current studies facing the problem of low classification accuracy [13, 14].

To solve the research difficulties, this paper proposed a character-level ensemble learning-based classification model. Five word embedding models, namely BERT, RoBERTa, XLNet, ERNIE and ELECTRA, were integrated. We used a metric learning based on Chinese corpus to accelerate the convergence of the model. In order to reduce the data imbalance problem, Focal Loss was introduced in training process. Finally, Soft Voting was used to ensemble the five models to improve the overall performance. The main contributions of this paper are as follows: (1) An ensemble model incorporating multiple character-level deep learning pre-training models was proposed for eligibility criteria text classification. (2) A combination strategy of focal loss and metric learning loss was proposed to solve data imbalance problem. (3) Experiment results demonstrated the effectiveness of the proposed model in eligibility criteria text classification by comparing with state-of-the-art methods.

## Related work

With the rapid development of deep learning [15], various short text classification methods have emerged. Kaljahi et al. [16] proposed the Any-gram kernel method to extract N-gram features from short textbooks and classify the text using bi-directional long- and short-term memory networks (Bi-LSTM). Convolutional neural networks (CNNs) were first used by Kim et al. [17] to solve text classification. Lee et al. [18] implemented merged recurrent neural networks (RNNs) and CNNs and proposed a new model for classifying short text. Hsu et al. [19] proposed a structure-independent gate-representation model for short text classification. In order to extract the features of the text in both temporal and spatial dimensions, Zhou et al. [20] introduced a two-dimensional maximum pooling operation in Bi-LSTM for the first time. In recent years, Google proposed the BERT model [21], which is based on Transformer [22], to improve feature extraction ability and to acquire context-sensitive bidirectional feature representations.

The research of clinical trial eligibility criteria classification has a positive effect on the design of eligibility criteria and effectively promote the recruitment of patient subjects. Zhang et al. promoted the matching of clinical trials for specific populations (such as HIV and pregnant women) through automatic classification of eligibility criteria of clinical trials [23]. In N2C2 2018 evaluation task [24], 288 complete longitudinal narrative medical records of diabetic patients and 13 pre-defined eligibility criteria were provided for identifying eligible patients. The top-ranked system in the evaluation used a rule-based method and achieved a micro F1 value of 0.91 [25]. In 2017, the American Society of Clinical Oncology (ASCO) studied the distribution of patients enrolled in clinical trials and the distribution of patients in the real world, and proposed that multiple screening criteria should be optimized and appropriately relaxed. These screening criteria include the inclusion of children in human cancer clinical trials The minimum age limit [26], the inclusion of HIV, hepatitis B or C infection [27], the inclusion of organ dysfunction, the second primary cancer or those with a previous history [28], and the inclusion of brain metastasis cancer patients [29] etc.

Metric learning [30, 31] aims to reduce or limit the distance between samples of the same class while increasing the distance between samples of different classes through training and learning. This approach has been widely used in various machine learning applications, including collaborative filtering, face recognition, and document retrieval [32–35]. Weinberger et al. proposed a large margin nearest neighbor (LMNN) approach [31] in learning a pull- and push-loss based metric to minimize the number of class impersonators. However, to the best of our knowledge, no existing work has been reported that focuses specifically on mitigating prediction uncertainty. When comparing feature representations of training data, Mandelbaum and Weinshall [36] measures model uncertainty through distance and it is inefficient for iterating over all training data. Metric learning is frequently applied to reduce model uncertainty in a text classification task.

## Methods

**Fig. 1:**
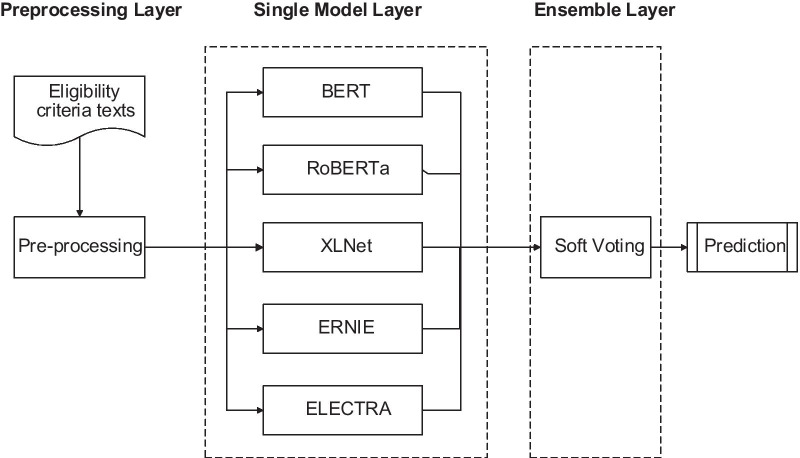
The framework of the ensemble learning-based model consists of a preprocessing layer, a single model layer integrating 5 pre-trained models including BERT, XLNet, ERNIE, RoBERTa, and ELECTRA, as well as an ensemble layer containing Soft Voting to output prediction result

The overall framework of our proposed ensemble learning-based model is shown in Fig. 1, which can be roughly divided into three layers: preprocessing layer, single model layer and model ensemble layer. After the input text pass through the preprocessing layer, it is converted from characters to numeric vectors for training in the next layer. Then, five single models based on different preprocessing methods are applied to train the vectors. Finally, the model ensemble is trained using the Soft Voting. The detailed structure of the model is presented in the next section.

### The architecture of single models

**Fig. 2:**
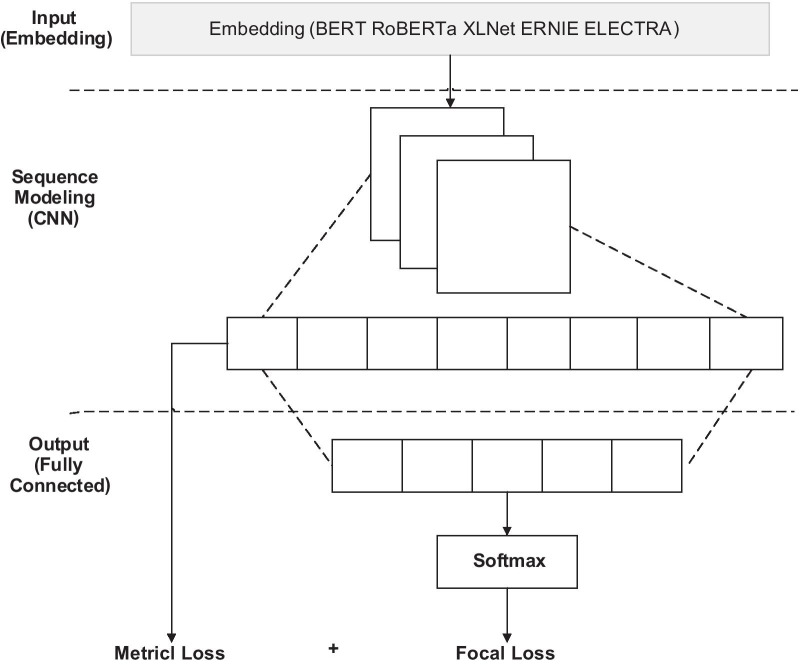
The overall architecture of the single models

The output of the five single models with a SoftMax function are as the input of the ensemble layer. To integrate the single models, the overall structure of the single models is designed, as shown in Fig. 2. The structure has three layers: (1) The input layer of each single model consists of five different pre-trained models, BERT, XLNet, RoBERTa, ERNIE, and ELECTRA. (2) The sequence modeling layer is implemented by a convolutional neural network (CNN) as well as a maximum pooling operation to extract the feature representation of word vectors. It utilizes three kernels with filter sizes of 3, 4, and 5. (3) The output consists of a full connection layer and a SoftMax operation. The loss function of the model is a combination of predicted Focal loss and metric loss. The output of the Sequence Modeling layer is considered as the representation of text and is used for the metric loss. The purpose here is to penalize large distance feature representations in the same category and small distance feature representations between different categories.

### Metric learning on text features

Making the feature distance between instances within a category much smaller than between instances is the purpose of learning the uncertainty of a text feature space. The feature distance can be either a European distance or a Manhattan distance. This goal can be achieved by training the embedding layer of the model through metric learning. Specifically, it can be expressed that ${r}_{i}$ and ${r}_{j}$ are the feature representations of instances *i* and *j*, respectively. Then the Euclidean distance between them is defined as $D\left({r}_{i},{r}_{j}\right)=\frac{1}{d}{\Vert {r}_{i}-{r}_{j}\Vert }_{2}^{2}$, where *d* is the dimension of the feature representation.

Assuming that a training data contains *n* categories, and ${S}_{k}$ represents an instance of data belonging to category *k*, the penalty for the distance between the feature representations of two instances of the same category is an intra-class loss, which can be formalized as Eq. (1).

1 $${L}_{intra}(k)=\frac{2}{{\left|{S}_{k}\right|}^{2}-\left|{S}_{k}\right|} \sum_{i,j\epsilon {S}_{k},i<j}D({r}_{i},{r}_{j})$$

$|S_{k} |$ represents the number of elements in set *S*_*k*_. The loss is the mean of all the distances between each possible pair in the same category set. The inter-class loss, as is formally defined as Eq. (2), ensures large feature distances between different category.

2 $${L}_{inter}\left(p,q\right)=\frac{1}{\left|{S}_{p}\right|*\left|{S}_{q}\right|} \sum_{i\epsilon {S}_{p}, j\epsilon {S}_{q}}\mathrm{max}(0,m-D({r}_{i},{r}_{j}))$$

*m* is the metric boundary constant that distinguishes two categories of data. If the feature distance between two data instances from different categories is greater than *m*, the inter-class loss is zero. Otherwise, the distance is subtracted from *m* as the loss. *m* represents the size of the inter-class feature distance and is set differently depending on word embedding methods. The overall metric loss function is defined in Eq. (3), which consists of inter-class and intra-class losses for all data categories.

3 $${L}_{metric}=\sum_{k=1}^{n}\left\{{L}_{intra}(k)+\lambda \sum_{i\ne k}{L}_{inter}(k,i)\right\}$$

*λ* is a pre-defined parameter to weight the importance of the intra- and inter-class losses. We set *λ* to 0.1 empirically in this paper.

### Loss function

**Fig. 3:**
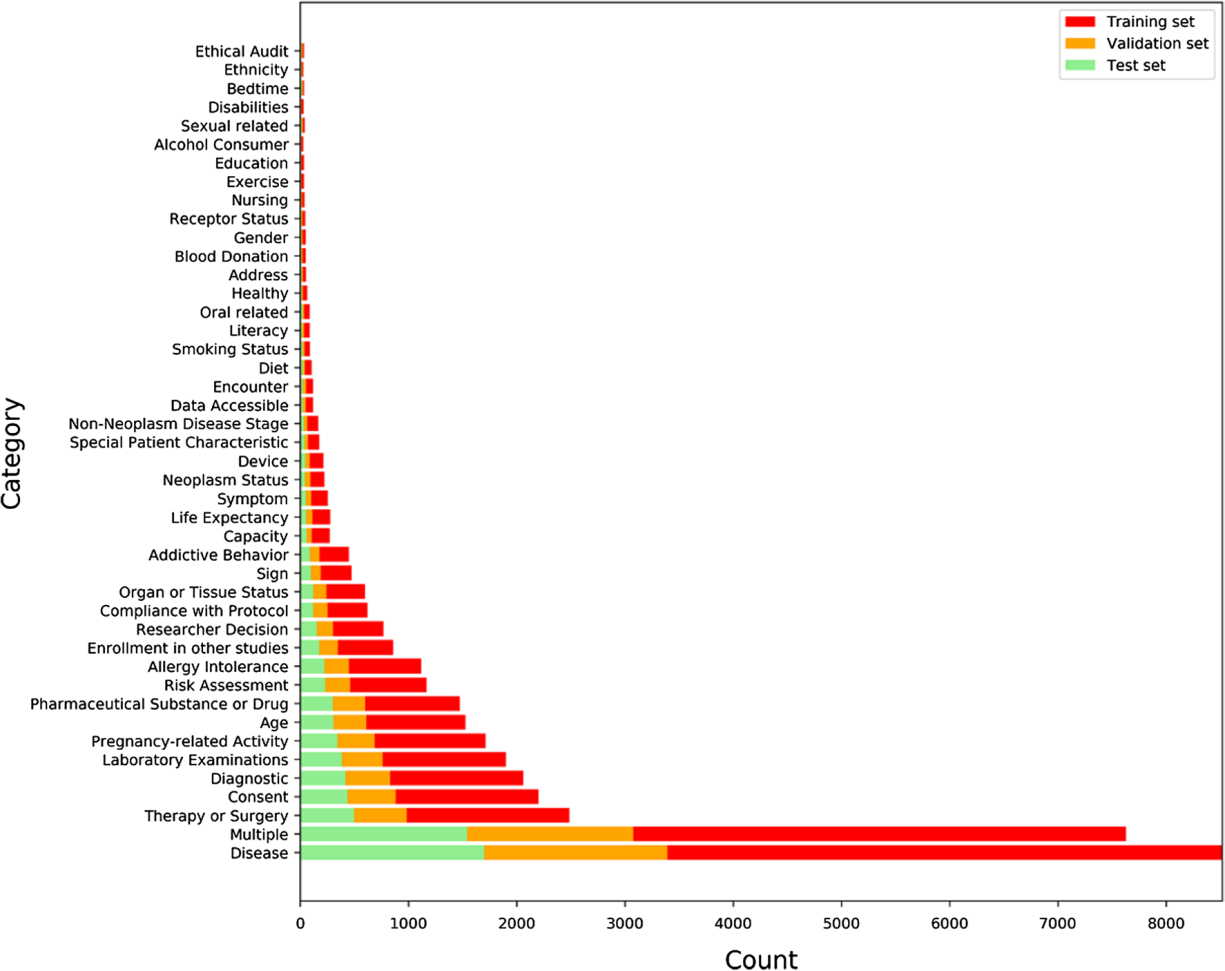
Histogram distributions of the training set, validation set, and test set. X-axis represents counts of data instances and Y-axis represents categories

Data imbalance problem commonly exits in eligibility criteria text and can be visualized from the distribution of data in training, validation, and test sets. Figure 3 shows the distribution of the count of instances in each category in the three datasets as introduced in experiments. There is a significant imbalance issue in the datasets for each category. The category with the highest count differs from the category with the lowest count by 8489 samples.

To reduce the data imbalance problem, focal Loss [37] is used as an alternative loss function during training. To show the advantage of Focal Loss, we compare Focal Loss with the formula for CE Loss (Cross Entropy Loss). Suppose the expression of ${p}_{t}$ is ${p}_{t}=\frac{{e}^{{x}_{t}}}{\sum_{j}{e}^{{x}_{j}}}$. ${x}_{t}$ is the score on category $t$, and ${p}_{t}$ is the prediction probability of an input sample on category $t$. The expression of CE Loss (Cross Entropy Loss) is calculated using Eq. (4).

4 $$CELoss={-\Sigma }_{i=1}^{n}{y}_{i}\mathrm{ log}({p}_{i})$$

${p}_{i}$ represents the predicted probability that the sample belongs to category $i$. Number of categories is represented by $n$. The formula for Focal Loss is shown in esquation (5), where *γ* is a predefined parameter and is set to 2 empirically in experiments.

5 $$Focal Loss= -{\left(1-{p}_{t}\right)}^{\gamma }\mathrm{log}({p}_{t})$$

${\left(1-{p}_{t}\right)}^{\gamma }$ is the modulation coefficient. The purpose of adding the coefficient is to make the model more focusing on difficult samples during training by reducing the weight of easy-to-classify samples. Specifically, when ${p}_{t}$ is close to 1, the modulation coefficient tends to 0, which means that the contribution to total loss is smaller. When ${p}_{t}$ tends to 0, the modulation factor is close to 1 and the loss is very less affected. In short, Focal Loss is a function to measure the contribution of difficult and easy-to-classify samples to summarize loss in data imbalance problem. The final loss function *L* during training consists of the metric learning loss as well as the Focal Loss, is expressed as Eq. (6).$\mu$ is the hyper-parameter and is empirically set to 1.

6 $$L=FocalLoss+{\mu L}_{metric}$$

### Model ensemble

In the last layer of the model, we obtain the SoftMax output of 5 single models in the previous layer, which is the probability that each data corresponds to 44 categories. It can be expressed as $M_{n,44}^{i}$, where $i$ represents the *i*-th single model and *n* represents the count of samples in the dataset. We use Soft Voting to perform model integration operations on these five base models. Specifically, the five sets of SoftMax outputs of each sample are averaged, and the corresponding subscript of the maximum probability value of the SoftMax result that obtained in the previous step is the final classification result $O_{n}$. The calculation through Soft Voting is expressed as Eq. (7).

7 $${O}_{n}=argmax\left(\frac{\sum_{i=1}^{5}{M}_{n.44}^{i}}{5}\right)$$

## Experiment

### Dataset

The dataset is from the third assessment task of the 2019 China Health Information Processing Conference (CHIP): the classification of short text of clinical trial eligibility criteria. The task is to classify irregular unstructured short eligibility criteria text into corresponding categories. The dataset contains a total of 44 categories of eligibility criteria text of clinical trials, including "disease", "multiple", and "Therapy or Surgery", with a total of 38,341 eligibility criteria text that have been manually annotated by human experts. The dataset is subdivided into a training set, a validation set, and a test set. The training set contains 22,962 text of eligibility criteria, while the validation and test sets contain 7,682 and 7,697 text, respectively. Examples of eligibility standard text and their labels are shown in Table 1. For example, the category corresponding to "Severe hearing or visual impairment" is "sign".

**Table 1:** Examples of eligibility criteria text and corresponding annotated categories

Eligibility criteria text	Category
年龄 > 80岁 (Age > 80)	Age
近期颅内或椎管内手术史 (recent intracranial or spinal canal surgery)	Therapy or surgery
血糖 < 2.7 mmol/L (Blood glucose < 2.7 mmol/L)	Laboratory examinations
性别不限,年龄18 ~ 70岁 (unlimited gender, age 18–70)	Multiple
合并造血系统或恶性肿瘤等严重原发性疾病(complicated with serious primary disease such as hematopoietic system or malignant tumor)	Disease
其他研究者认为不适合参加本研究的患者 (patients that unsuitable for this study considered by other investigators)	Researcher decision
预期生存超过12周 (expected survival over 12 weeks)	Life expectancy
男、女不限 (male or female)	Gender

### Experiment setting-up

In the experiments, the random seed is set to 0 to ensure that results are reproducible and easy to compare between different model performances. The parameters of each pre-trained model are kept unchanged, the learning rate is set to 2 × 10^–5^, and the batch size is 128. Each single model is trained with regularization to prevent overfitting. Adam is used as the optimizer, and the Tesla K80 graphics card is used for training with memory size as 12.5G. 5 single models are trained separately due to limited memory. The epoch for each model is set to 12. More specifically, the Chinese pre-training models BERT^1^, RoBERTa^2^, XLNet^3^, ERNIE^4^, and ELECTRA^5^ are all pre-trained using Chinese wikis as well as encyclopedias, news, and quizzes, with a total word count of 5 billion and a text size of about 10G. The time cost of ensemble learning is about 8 h. The time cost consists mainly of single-model training time, of which 1.5 h are required per single model. The model is implemented based on the PyTorch framework.

### Evaluation metrics

In order to evaluate the performance of our model, in addition to the Macro F1-score specified by the CHIP2019 evaluation task, we used three extra metrics commonly used in deep learning classification tasks: Accuracy, Precision, and Recall. Macro F1-score is a parameter metric that reflects model validity and stability. The formula for these four evaluation metrics are shown in Eqs. (8)–(11).

8 $$Accuracy=\frac{TP+TN}{TP+TN+FP+FN}$$

9 $$Precision=\frac{TP}{TP+FP}$$

10 $$Recall=\frac{TP}{TP+FN}$$

11 $$F1\left(Macro\right)=\left(\frac{1}{n}\right)\sum \frac{2\times Precision\times Recall}{Precision+Recall}$$

$TP$ (True Positive) is the count of cases that are correctly predicted as *True*. $FP$ (False Positive) is the count of cases that are wrongly predicted as *True*.$FN$ (False Negative) is the count of cases that are model wrongly predicts as *False*. $TN$(True Negative) is the count of cases that are correctly predicted as *False*. *n* denotes the count of categories, as 44 in this paper.

## Results

In order to evaluate the validity of our proposed model, we compared our ensemble model with other deep learning-based classification models including TextCNN, TextRNN, TextRCNN, FastText, and Transformer models. The result, as shown in Table 2, presented that the macro F1-scores of the models were between 0.6721 by transformer and 0.7704 by TextRCNN. In order to verify the effect of model ensemble, 5 single models including BERT, XLNet, ERNIE, RoBERTa and ELECTRA were implemented as benchmarks for comparison. As shown in the same table, XLNet and RoBERTa achieved high performances among the single models as 0.803 and 0.7992, respectively. Our ensemble learning-based model using metric learning achieved the best performance 0.8167, with an average increase of 2.58% compared to the single models. The performance of our model exceeded the best performed model in CHIP 2019 Task 3 challenge as state-of-the-art with a macro F1-score of 0.8095, while the second with 0.8080 and the third with 0.8075. Finally, we performed a t-test on the performance of the ensemble learning-based model versus the performance of the other five single models. The p-value was 2.152e-07, indicating that the performance of our model had a significant improvement compared with the single model.

**Table 2:** The performances of our model and baseline models on the same dataset

Model	Accuracy	Precision	Recall	Macro F1
TextCNN	0.8256	0.8074	0.7538	0.7696
TextRNN	0.8094	0.7262	0.7369	0.7258
TextRCNN	0.8256	0.7894	0.7678	0.7704
FastText	0.8116	0.7732	0.7268	0.7385
Transformer	0.7934	0.7545	0.6469	0.6721
BERT	0.8385	0.8055	0.7980	0.7973
XLNet	0.8508	0.8164	0.8011	0.803
ERNIE	0.8382	0.8035	0.7969	0.7952
RoBERTa	0.8439	0.7929	0.8215	0.7992
ELECTRA	0.8324	0.7935	0.791	0.7862
Our model	0.850	0.825	0.821	0.8167

### The impact of metric learning on feature representation

The impact of metric learning on feature representation was analyzed. As shown in Table 3, the second column presented performance of models trained without metric learning and the third column denoted performance of models with metric learning. From the result, the model ELECTRA pre-training model achieved the highest performance with an increasing rate of 1.34% when using metric learning, while model RoBERTa obtained the least macro F1 score improvement as 0.52% when using metric learning. Overall, the increasing rate of macro F1 score was 0.95% on average when using metric learning. In addition, the macro F1 score performance of the 5 single models under different loss function was also compared. As shown in the Table 4, the performance of the models with Focal Loss is higher than that with Cross Entropy Loss for every model. The model pre-trained with ERNIE had the largest performance improvement when using Focal Loss.

**Table 3:** Performance comparison of all single models with or without metric learning using macro F1 score (margin parameter *m* = 0.1)

Model	Without metric learning	With metric learning	Increase rate (%)
BERT	0.7880	0.7973	1.18
XLNet	0.7983	0.8030	0.59
RoBERTa	0.7951	0.7992	0.52
ERNIE	0.7865	0.7952	1.11
ELECTRA	0.7758	0.7862	1.34

**Table 4:** Performance comparison of all single models with cross entropy loss or focal loss functions using macro F1 score

Model	Cross entropy loss	Focal loss
BERT	0.7902	0.7973
XLNet	0.7987	0.8030
RoBERTa	0.7959	0.7992
ERNIE	0.7868	0.7952
ELECTRA	0.7804	0.7862

### The impact of training data volume on model performance

**Fig. 4:**
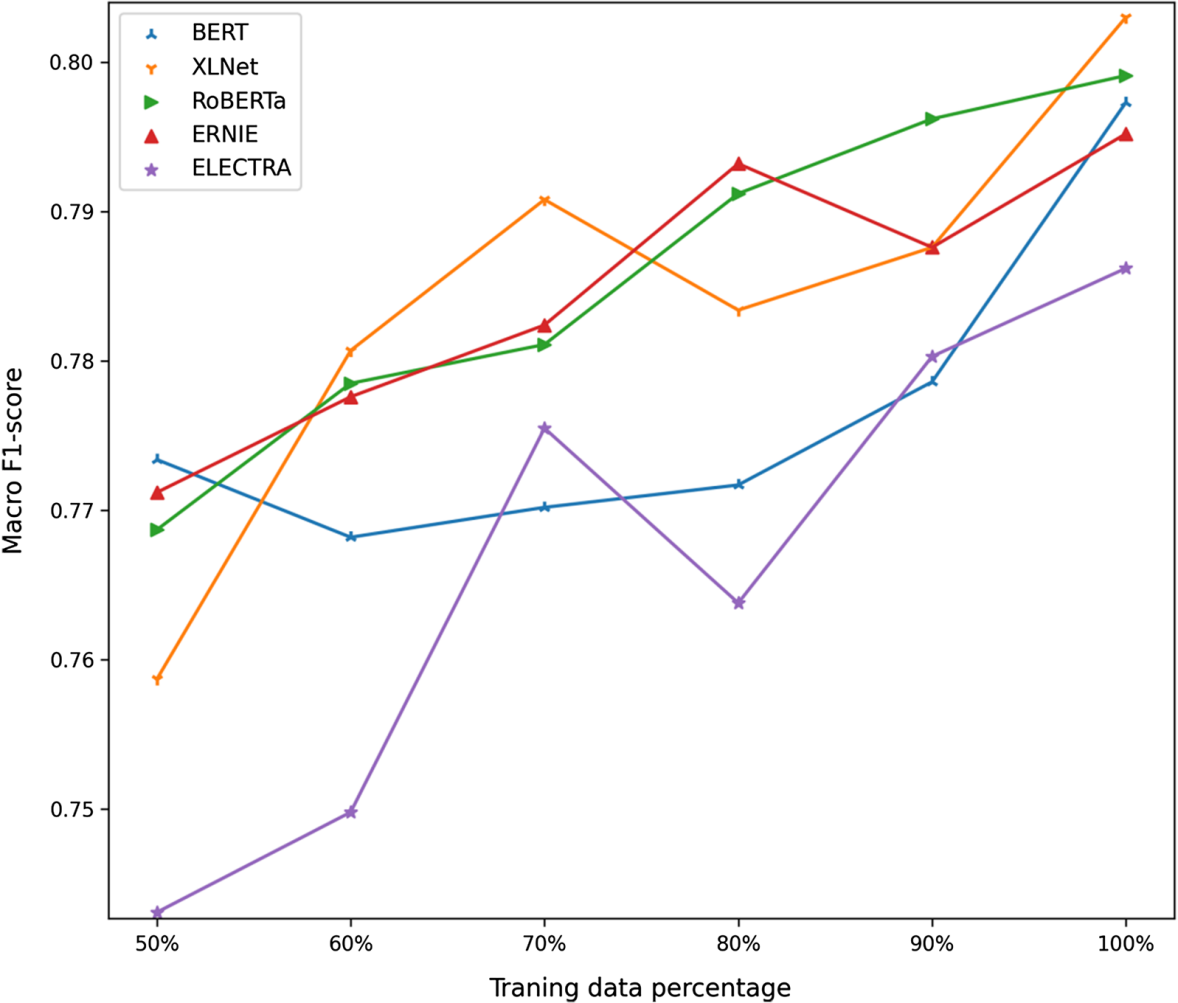
Performance of single models based on BERT and XLNet pre-training models under different percentages of data volume

To test the impact of training data volume on model performance, we keep the training set unchanged and randomly reduce the amount of data in the training set by 10%, 20%, 30%, 40%, and 50%. The experiment was performed separately on BERT and XLNet models. The results are shown in Fig. 4. Compared with the results of the whole data, the performance of these two models under the reduced data volume was significantly lower than the performance on the whole data. Among them, by reducing the data to 50%, the F1 score of the BERT model reduced by 1.32%, while that of XLNet model reduced by 5.24%.

## Discussion

Through experiment analysis, there were two constraints that limited the training and final performance of our model. (1) The selection of hyper-parameters in metric learning. *m* was the metric boundary constant that distinguished the data. $\lambda$ was a pre-defined parameter to weight the importance of the intra- and inter-class losses. In the experiment, we found that different parameter (*m* and $\lambda$) values had different effects on the performance of different models. Therefore, effort was needed to adjust the parameters of each model as it affected the efficiency and performance of the models. (2) Insufficient training data. From the experiment analysis, it can be found that insufficient training data may be an important factor in limiting the model performance.

In addition, the eligibility criteria text had not been preprocessed before models training due to specific difficulties. For example, many special symbols/characters in sentences existed, such as special expression (symbols of numbers, operators, or units), stop words, traditional Chinese characters, and full-width characters. Thus, text data preprocessing was needed to improve the performance of the classifiers.

Ensemble learning is a machine learning framework whose main idea was to combine multiple base models and to fuse potential differences learned by different single models to improve the generalization ability of the overall model. In addition to the Soft Voting method used in the experiments, there were two other algorithms, AdaBoost and Stacking, tested. However, due to insufficient training data, each single model was easily overfitting, so the Voting algorithm was experimentally applied to outperform the other two algorithms.

Two directions, as data and model, were the subsequent breakthroughs to improve the performance of our model. The short eligibility criteria text had irregular and low word count characteristics, so it did not provide sufficient information. Therefore, effective data enhancement methods could be applied on the short text data to enhance the textual features for improvement purposes. Secondly, for textual data in the medical domain, pre-training the model through medical corpus might help to enhance the stability of the model.

## Conclusion

Automated classification of clinical trial eligibility criteria text is a fundamental and critical procedure in clinical target population recruitment. This research proposed an ensemble learning-based model by integrating deep learning methods including BERT, ERNIE, XLNet, ELECTRA, and RoBERTa. The model was compared with a list of baseline deep learning models on a publicly available standard data set. The results demonstrated that our proposed model outperformed baseline models with 2.58% improvement on average. The utilization of metric learning effectively improved the performance of single models. The Focal Loss was more suitable for eligibility criteria text classification with data imbalance issue.

## Data Availability

Data are provided by Guangdong Mental Health center and it cannot be shared with other research groups without necessary permission.

## Abbreviations

NLP: natural language processing
LSTM: long short-term memory
BILSTM: bi-directional long short-term memory
CNN: convolutional neural network
RNN: recurrent neural network
BERT: bidirectional encoder representations from transformers
NSP: next sentence prediction
RoBERTa: a robustly optimized BERT pretraining approach
WWM: whole word masking
ERNIE: enhanced representation through knowledge integration
CHIP: China Health Information Processing

## Footnotes

1
https://huggingface.co/bert-base-chinese.

2
https://github.com/brightmart/roberta_zh.

3
https://github.com/ymcui/Chinese-XLNet.

4
https://github.com/PaddlePaddle/ERNIE.

5
https://github.com/ymcui/Chinese-ELECTRA.
